# Vitamin D status and its association with metabolic syndrome and cardiometabolic markers in postmenopausal women: a population-based study

**DOI:** 10.20945/2359-4292-2026-0085

**Published:** 2026-07-27

**Authors:** Laura Alves Cota e Souza, Thaís Teixeira Silva Diniz, Angélica Alves Lima

**Affiliations:** 1 Programa de Pós-graduação em Ciências Farmacêuticas (CiPharma), Escola de Farmácia, Universidade Federal de Ouro Preto, Ouro Preto, MG, Brasil; 2 Departamento de Análises Clínicas (DEACL), Escola de Farmácia, Universidade Federal de Ouro Preto, Ouro Preto, MG, Brasil

**Keywords:** Vitamin D, postmenopausal women, metabolic syndrome, anthropometric parameters, biochemical markers

## Abstract

**Objective:**

To investigate the association between serum vitamin D levels, metabolic syndrome (MetS), and cardiometabolic markers in postmenopausal women from a population-based sample in Brazil.

**Subjects and methods:**

This cross-sectional study included 351 women aged 40 to 64 years with at least 12 months of amenorrhea. Serum 25-hydroxyvitamin D was measured by chemiluminescence and categorized into quartiles. MetS was defined according to the Joint Interim Statement criteria. Anthropometric, blood pressure, lipid profile, insulin, calcium, and phosphorus measures were also assessed.

**Results:**

Hypovitaminosis D (vitamin D <30 ng/mL) was observed in 66.4% of participants. Women in the highest quartile (≥31.0 ng/mL) had significantly more favorable metabolic profiles than those in the lowest quartile (<21.0 ng/mL), including lower BMI, waist circumference, insulin, and triglycerides, and higher HDL cholesterol. Vitamin D levels <21.0 ng/mL were associated with a 2.27-fold increased risk of MetS (95% CI: 1.21-4.24; p = 0.010).

**Conclusion:**

Higher vitamin D levels are associated with more favorable metabolic profiles and a reduced risk of MetS in postmenopausal women. Adequate vitamin D status may play a preventive role in mitigating metabolic alterations during postmenopause.

## INTRODUCTION

Vitamin D (VitD) is a fat-soluble prohormone that is primarily synthesized in the skin upon exposure to ultraviolet B radiation and, to a lesser extent, obtained from dietary sources (^1^,^2^). The circulating form, 25-hydroxyvitamin D (25[OH]D)], reflects VitD status and serves as a precursor to the active metabolite, 1,25-dihydroxyvitamin D [1,25(OH)_2_D], which regulates calcium and phosphate balance and supports bone health (^2^,^3^).

Beyond its established role in skeletal health, VitD exerts pleiotropic effects on multiple tissues that express VitD receptors and hydroxylation enzymes, influencing immune, cardiovascular, and metabolic pathways (^4^-^6^). Hypovitaminosis D represents a significant global public health concern, largely attributable to factors such as reduced sun exposure, darker skin pigmentation, aging, and obesity (^7^,^8^). Although some interventional studies have reported limited or no effects of VitD supplementation on glucose or lipid metabolism (^9^,^10^), VitD deficiency has been associated with hypertension, insulin resistance, dyslipidemia, and metabolic syndrome (MetS) (^4^,^6^,^11^). Moreover, recent clinical and meta-analytic studies have explored the impact of dietary and VitD-related factors on cardiometabolic outcomes, highlighting potential links between nutritional interventions and metabolic health (^12^-^14^).

Among postmenopausal women, estrogen decline may decrease hepatic 25-hydroxylase activity and reduce cutaneous VitD synthesis (^15^,^16^). These hormonal and physiological alterations, combined with insufficient sun exposure and dietary inadequacy, render this group particularly susceptible to VitD deficiency (^14^). Indeed, a Brazilian study reported that approximately 60% of older women exhibit suboptimal VitD concentrations (^17^).

In addition to their susceptibility to hypovitaminosis D, postmenopausal women are at elevated risk for MetS - a cluster of cardiometabolic abnormalities that includes central obesity, hypertension, dyslipidemia, and insulin resistance - and consequently have a higher likelihood of developing type 2 diabetes and cardiovascular disease (^15^,^18^). The coexistence of hypovitaminosis D and MetS may have synergistic impact, as hormonal decline, increased visceral adiposity, and inflammatory changes during menopause can simultaneously impair VitD metabolism and exacerbate adverse cardiometabolic profiles (^19^). This intersection underscores the importance of investigating the relationship between VitD status and MetS in this population.

Therefore, this population-based study aimed to examine the association between serum VitD levels, the presence of MetS, and cardiometabolic risk markers in postmenopausal women from Brazil. We hypothesized that lower VitD concentrations would be associated with a higher prevalence of MetS and an unfavorable metabolic profile.

## SUBJECTS AND METHODS

### Study participants and data collection

A total of 377 participants were recruited based on practical considerations and availability during the study period. Eligible participants were women aged 40-64 years who had experienced at least 12 consecutive months of amenorrhea. Participants were randomly selected from a registry of users of the Brazilian Unified Health System in Ouro Preto (Minas Gerais State, Brazil). Women within the target age range were identified from this registry and invited to participate via telephone calls or home visits. Additionally, eligible women were approached and recruited by community health agents, healthcare professionals, and members of the research team. Women who reported the current use of VitD supplements or who declined to participate in the interviews or assessments were excluded.

Data collection was carried out at primary healthcare units and facilities at the Federal University of Ouro Preto. Structured interviews were administered using standardized questionnaires that captured sociodemographic, reproductive, and lifestyle variables. This study was approved by the Research Ethics Committee of the Federal University of Ouro Preto under protocol number 56312916.8.0000.5150. All participants provided written informed consent.

### VitD and other laboratory tests

Blood samples were collected after participants had fasted for 12-14 hours, abstained from alcohol for 72 hours, and avoided physical activity for 24 hours. The data collection period spanned more than a year, covering all seasons, which helped minimize the potential impact of seasonal variations in sunlight exposure on serum VitD concentrations. Serum 25-hydroxyvitamin D [25(OH)D] and insulin levels were measured via chemiluminescence using the Access 2 Immunoassay System^®^ (Beckman Coulter). The lipid profile (total cholesterol, HDL-c, LDL-c, and triglycerides), fasting glucose, calcium, and phosphorus levels were determined using the Cobas Integra^®^ 400 Plus analyzer (Roche). Derived indices included non-HDL cholesterol, the Homeostasis Model Assessment of Insulin Resistance (HOMA-IR) (^20^), and the quantitative insulin sensitivity check index (QUICKI) (^21^), calculated using **Equations 1-3:**


(1) non -HDLc=TC(mg/dL)-HDLc(mg/dL)

(2) $$\text{ HOMA }-\mathrm{IR}=(\text{ fasting blood glucose }\times 0.0555)\times \frac{\text{ insulin }}{22.5}$$

(3) $$\text{ QUICK }=\frac{1}{\text{ loglog Insulin }\left(\frac{µUI}{mL}\right)+\left(\frac{mg}{dL}\right)}$$

MetS was defined based on the harmonized criteria established by the joint interim statement (^22^).

### Blood pressure and anthropometric measurements

Blood pressure was measured using a Bioland^®^-3005 digital wrist monitor, following the manufacturer’s instructions. Anthropometric measurements included weight, height, waist circumference, and body fat percentage. Weight and body fat were obtained using a Tanita^®^ digital bioimpedance scale (Model 2204; precision = 100 g; capacity = 150 kg), with participants standing barefoot, and upright in the center of the scale. Height was measured using a wall-mounted stadiometer (precision = 0.1 cm; maximum = 2.0 m) with participants standing erect, feet together, arms resting at their sides, and the head positioned in the Frankfurt plane. Waist circumference was measured with a non-elastic measuring tape at the midpoint between the lowest rib and the iliac crest; when this landmark was difficult to locate, the measurement was taken at the umbilical level.

The following indices were then calculated **(Equations 4-6):**


(4) $$\text{ BMI }=\frac{\text{ Weight }(\mathrm{kg})}{[\text{ Height }(m){]}^{2}}$$

(5) $$\text{ WHtR }=\frac{\text{ Waist circumference }(\mathrm{cm})}{\text{ Altura }(\mathrm{cm})}$$

(6) $$\text{ Conicity index }=\frac{\text{ Waist circumference }(m)}{0.109\sqrt{\frac{\text{ Weight }(\mathrm{kg})}{\text{ Height }(m)}}}$$

### Statistical analysis

Data were coded and double-entered using EpiData to ensure accuracy. Statistical analyses were performed using SPSS version 20.0 (IBM Corp.). Serum 25(OH)D concentrations were categorized into quartiles. Binary logistic regression was used to estimate unadjusted and adjusted odds ratios (OR) and 95% confidence intervals (CI) for MetS across VitD quartiles, using the highest quartile (Q4: ≥ 31.0 ng/mL) as the reference group.

Categorical variables were compared using Pearson’s chi-square test. The Kolmogorov-Smirnov test was used to assess the normality of continuous variables. Because most variables were non-normally distributed, data were expressed as medians and interquartile ranges and compared using the Kruskal-Wallis test. Post hoc pairwise comparisons were performed with the Bonferroni correction to account for multiple testing. Effect sizes (η^2^h) for the Kruskal-Wallis tests were calculated according to Tomczak and Tomczak (2014), using the formula η^2^h = (H - k + 1)/(n - k), where H is the Kruskal-Wallis test statistic, k is the number of groups, and n is the total sample (^23^). A post-hoc power analysis was performed using G*Power software (F tests, analysis of variance: fixed effects, omnibus, one-way) with a minimum power of 80% and a significance level of 0.05. Only participants with complete data and no missing values were included in the final analysis. A *p*-value < 0.05 was considered statistically significant.

## RESULTS

**Figure 1:**
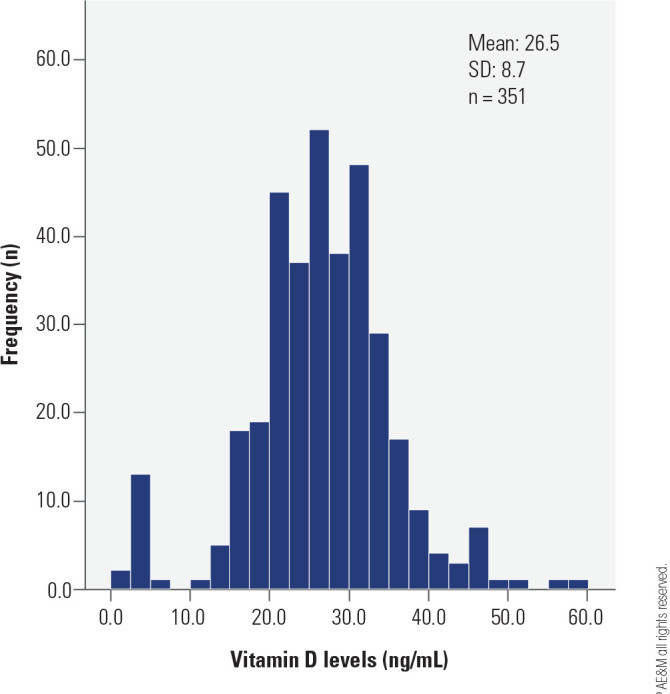
Distribution of serum 25(OH)D levels among postmenopausal participants (n = 351).

A total of 377 postmenopausal women were initially recruited. After excluding 14 participants who reported current use of vitD supplements and 12 with missing data, 351 women were included in the final analysis. The mean serum 25(OH)D concentration in the sample was 26.5 ± 8.7 ng/mL, with a median of 26.0 ng/mL and values ranging from 2.4 to 59.0 ng/mL (**Figure 1**). The distribution of serum VitD levels was approximately normal, with most participants clustering between 20 and 30 ng/mL, reflecting the predominance of suboptimal VitD status in this population. Hypovitaminosis D, defined as a concentration below 30.0 ng/mL, was observed in 66.4% of participants (n = 233). For analysis, participants were categorized into quartiles based on their serum 25(OH)D levels: Q1 (< 21.0 ng/mL), Q2 (21.0-26.9 ng/mL), Q3 (27.0-30.9 ng/mL), and Q4 (≥ 31.0 ng/mL).

Across all quartiles, most participants were between 50-60 years of age, were married or living with a partner, had a family income greater than twice the minimum wage, and reported current medication use. Smoking and alcohol consumption were infrequent, while 48.4% of the sample (n = 170) reported regular physical activity. The median age at menopause was 48 years, and the median time since menopause was six years. Hypertension and neuropsychiatric conditions were the most frequently reported comorbidities. There were no statistically significant differences in sociodemographic or behavioral variables between vitD quartiles (**Table 1**).

**Table 1:** Sociodemographic and behavioral variables of the participants according to quartiles of vitamin D

Variable	Quartile of Vitamin D					p-value	χ^2^
	Q1 (n=76) < 21.0 ng/mL	Q2 (n=108) 21.0-26.9 ng/mL	Q3 (n=76) 27.0 - 30.9 ng/mL	Q4 (n=91) ≥ 31.0 ng/mL	Total (n=351)		

	n (%) or median (1º and 3º quartiles)						
Age (years)	56.0	55.0	55.0	55.0	55.0	0.407	0.889
	(53.0-59.0)	(52.0-58.0)	(52.0-58.0)	(52.0-59.0)	(52.0-58.0)		
40-49	8 (10.5)	11 (10.2)	9 (11.8)	10 (11.0)	38 (10.8)	0.185	12.541
50-54	17 (39.0)	38 (35.2)	25 (32.9)	35 (38.5)	115 (32.8)		
55-60	38 (50.0)	43 (39.8)	34 (44.7)	26 (28.6)	141 (40.2)		
61-65	13 (17.1)	16 (14.8)	8 (10.5)	20 (22.0)	57 (16.2)		
Married/living with a partner	49 (64.5)	66 (61.1)	50 (65.8)	53 (58.2)	218 (62.1)	0.743	1.242
Family income (MW)^*^							
0,00 - 1,00	18 (23.7)	27 (25.0)	17 (22.4)	21 (23.1)	83 (23.6)	0.987	2.231
1,01 - 2,00	22 (28.9)	28 (25.9)	23 (30.3)	22 (24.2)	95 (27.1)		
> 2,00	32 (42.1)	43 (39.8)	30 (39.5)	41 (45.1)	146 (41.6)		
NA	4 (5.3)	10 (9.3)	6 (7.9)	7 (7.7)	27 (7.7)		
Use of medicines	62 (81.6)	75 (69.4)	50 (65.8)	69 (75.8)	256 (72.9)	0.117	5.894
Current smoke	9 (11.8)	13 (12.0)	9 (11.8)	14 (15.4)	45 (12.8)	0.867	0.725
Alcohol use	1 (1.3)	7 (6.5)	1 (1.3)	2 (2.2)	11 (3.1)	0.116	5.905
Regular physical activity	33 (43.4)	52 (48.1)	39 (51.3)	46 (50.5)	170 (48.4)	0.757	1.184
Age of menopause (years)	48.0	48.0	48.0	48.0	48.0	0.819	0.927
	(45.0-51.3)	(43.0-51.0)	(43.0-51.0)	(44.0-51.0)	(44.0-51.0)		
Time of menopause (years)	6.5	5.0	6.0	7.0	6.0	0.711	1.379
	(2.8-10.0)	(2.0-11.0)	(3.0-9.0)	(3.0-10.0)	(2.0-10.0)		
History of diseases							
Hypertension	37 (48.7)	35 (32.4)	23 (30.3)	41 (45.1)	136 (38.7)	0.032	8.821
Neuropsychiatric disease	22 (28.9)	27 (25.0)	22 (28.9)	36 (39.6)	107 (30.5)	0.155	5.240
Gastritis	22 (28.9)	23 (21.3)	19 (25.0)	22 (24.2)	86 (24.5)	0.699	1.427
Thyroid disorders	11 (14.5)	20 (18.5)	10 (13.2)	20 (22.0)	61 (17.4)	0.419	2.828

* The minimum wage (MW) during data collection varied from BRL 724.00 to BRL 1,100.00. NA = participant did not answer; the statistical analysis did not show significant differences among the quartiles.

Anthropometric and metabolic parameters varied significantly according to vitD status (**Table 2**). Women in the highest quartile (Q4) showed more favorable anthropometric profiles than those in the lowest quartile (Q1). Specifically, they presented significantly lower values for weight (63.8 vs. 72.2 kg; *p* = 0.009), body mass index (25.6 vs. 28.5 kg/m^2^; *p* = 0.005), body fat percentage (34% vs. 38%; *p* = 0.013), waist circumference (88 vs. 98 cm; *p* = 0.002), waist-to-height ratio (0.57 vs. 0.61; *p* = 0.006), and conicity index (1.27 vs. 1.31; *p* = 0.027). Significant differences between Q3 and Q1 were also observed for waist-to-height ratio (0.57 vs. 0.61; *p* = 0.028) and conicity index (1.27 vs. 1.31; *p* = 0.040).

**Table 2:** Anthropometric, blood pressure, and biochemical data of the participants according to quartiles of vitamin D

	Quartile of Vitamin D				Statistics		
Variable	Q1 (n=76) < 21.0 ng/mL	Q2 (n=108) 21.0-26.9 ng/mL	Q3 (n=76) 27.0-30.9 ng/mL	Q4 (n=91) ≥ 31.0 ng/mL	*p*-value	Test Statistics (H)	Effect size (η^2^)
	Median (1º - 3º quartile)						
**Anthropometric variables**							
Weight (kg)	72.2 (63.6-79.8)	67.1 (59.5-77.5)	66.9 (62.2-74.5)	63.8 (58.4-75.9)ª	0.016	10.363	0.021
BMI (kg/m^2^)	28.5 (25.8-31.7)	26.8 (24.4-31.3)	26.9 (24.3-30.2)	25.6 (23.1-28.8)ª	0.009	11.481	0.024
Body fat (%)	38.0 (34.0-42.0)	36.0 (31.8-41.0)	36.0 (30.0-40.3)	34.0 (29.5-39.0)ª	0.023	9.548	0.019
WC (cm)	98.0 (88.5-101.0)	91.0 (82.0-101.3)	90.5 (83.8-99.0)	88.0 (82.0-98.0)ª	0.003	13.851	0.031
WHtR	0.61 (0.56-0.64)	0.58 (0.53-0.65)	0.57 (0.52-0.62)ª	0.57 (0.51-0.62)ª	0.006	12.502	0.027
Conicity index	1.31 (1.26-1.37)	1.28 (1.23-1.34)	1.27 (1.22-1.33)ª	1.27 (1.22-1.34)ª	0.016	10.340	0.021
**Blood pressure**							
Systolic	129.5 (120.0-140.0)	132.5 (120.8-145.3)	130.0 (121.0-142.3)	130.0 (122.0-144.5)	0.815	0.943	0.000
Diastolic	82.0 (78.0-90.0)	84.0 (78.0-90.3)	82.0 (77.0-88.3)	83.0 (77.5-89.5)	0.815	0.945	0.000
**Biochemical variables**							
Triglycerides (mg/dL)	137.5 (102.0-196.0)	115.0 (82.3-169.8)	116.0 (89.5-159.0)	109.0 (75.0-144.0)ª	0.004	13.080	0.029
TC (mg/dL)	211.0 (186.8-246.3)	218.5 (192.5-244.5)ª	214.0 (196.0-236.3)	207.0 (174.5-239.0)	0.285	3.789	0.002
HDLc (mg/dL)	48.5 (39.8-60.0)	55.5 (46.0-67.3)ª	54.5 (45.8-65.0)	59.0 (48.5-67.5)ª	0.001	17.471	0.042
LDLc (mg/dL)	128.6 (113.3-161.2)	137.6 (111.5-157.1)	129.0 (113.7-161.6)	124.6 (97.4-155.3)	0.456	2.606	0.000
Non-HDLc (mg/dL)	168 (138.0-190.0)	160.5 (133.0-182.3)	157.0 (135.8-180.3)	149.0 (116.5-179.0)	0.082	6.705	0.011
FBG (mg/dL)	94.0 (88.8-106.0)	93.0 (87.0-104.0)	92.5 (87.0-101.3)	89.0 (84.0-99.0)	0.152	5.285	0.007
Insulin (µUI/mL)	7.9 (4.8-11.9)	7.4 (4.6-10.6)	5.9 (4.4-10.1)	5.8 (4.2-7.9)ª	0.015	10.516	0.022
HOMA-IR	1.68 (1.11-3.00)	1.64 (1.02-2.57)	1.41 (1.05-2.71)	1.31 (0.89-1.74)ª	0.015	10.506	0.022
QUICKI	0.35 (0.32-0.38)	0.35 (0.33-0.38)	0.36 (0.33-0.38)	0.37 (0.35-0.39)ª	0.015	10.503	0.022
Calcium (mg/dL)	9.7 (9.3-10.2)	9.6 (9.3-9.9)	9.7 (9.4-10.1)	9.7 (9.4-10.0)	0.622	1.769	0.000
Phosphorus (mg/dL)	3.8 (3.5-4.2)	3.8 (3.4-4.1)	3.8 (3.5-4.1)	3.9 (3.5-4.2)	0.133	5.588	0.007

a *p* < 0.05 compared with Q1. BMI: body mass index; WC: waist circumference; WHtR: waist-to-height ratio; TC: total cholesterol; HDL-C: high-density lipoprotein cholesterol; LDL-C: low-density lipoprotein cholesterol; FBG: fasting blood glucose; HOMA-IR: homeostasis model assessment of insulin resistance; QUICKI: quantitative insulin sensitivity check index.

No statistically significant differences in blood pressure were found between the quartiles (**Table 2**). However, participants in Q4 exhibited significantly higher median HDL-C levels (59 vs. 49 mg/dL; *p* < 0.001) and QUICKI values (0.37 vs. 0.35; *p* = 0.015), along with lower triglyceride levels (109 vs. 138 mg/dL; *p* = 0.002), insulin concentrations (5.8 vs. 7.9 µIU/mL; *p* = 0.021), and HOMA-IR (1.31 vs. 1.68; *p* = 0.015) when compared with Q1. A significant difference in HDL-C was also found between Q2 and Q1 (56 vs. 49 mg/dL; *p* = 0.016).

Participants diagnosed with MetS had significantly lower serum VitD levels compared with those without the condition (25.4 ± 8.0 vs. 27.3 ± 9.1 ng/mL; *p* = 0.041). In the multivariate logistic regression analysis, women in the lowest VitD quartile (Q1) had a significantly higher likelihood of having MetS than those in the highest quartile (Q4), both in the unadjusted model (OR = 2.27; 95% CI: 1.21-4.24; *p* = 0.010) and after adjustment for age, smoking, and physical activity (OR = 2.15; 95% CI: 1.14-4.05; *p* = 0.019). In addition, a trend toward an increased MetS risk was also observed in quartiles Q2 and Q3, although these associations did not reach statistical significance (**Table 3**). The power analysis indicated that the minimum detectable effect size was Cohen’s f = 0.177, corresponding to approximately 80% power to detect small-to-moderate differences across VitD quartiles.

**Table 3:** Odds ratios (OR) and 95% confidence intervals (CI) for metabolic syndrome according to serum vitamin D quartiles, unadjusted and adjusted for age, smoking, and physical activity

Vitamin D quartile	OR	95% CI	*p*-value	Adjusted OR^*^	95% CI	*p*-value
Q1 (< 21.0 ng/mL)	2.27	(1.21-4.24	0.010	2.15	1.14-4.05	0.019
Q2 (21.0 - 26.9 ng/mL)	1.55	0.87-2.76	0.137	1.55	0.86-2.79	0.143
Q3 (27.0 - 30.9 ng/mL)	1.74	0.93-3.26	0.082	1.81	0.96-3.43	0.068
Q4 (≥ 31.0 ng/mL)	1	-	-	1	-	-

* Adjusted for age, physical activity, and smoking.

## DISCUSSION

We found that a high percentage (60%) of postmenopausal women from Ouro Preto (Minas Gerais State, Brazil) had serum VitD levels below 30.0 ng/mL. The high prevalence of hypovitaminosis D is a well-documented issue in various geographic regions, including Brazil (^7^,^24^). Because older adults spend more time indoors and have a reduced dermal capacity to synthesize VitD, they are at an increased risk of hypovitaminosis D (^25^). Additionally, hypoestrogenism, which is common after menopause, can contribute to a decline in VitD levels (^26^). Several studies have reported that VitD deficiency affects over 90% of individuals, depending on the studied population (^7^). A systematic review of 32 studies worldwide found that 77.4% (n = 16,440) of postmenopausal women had low serum VitD concentrations (<30 ng/mL), with the prevalence of hypovitaminosis D ranging from 29% in the United States to 99.4% in China (^27^). Consistent with our findings, another Brazilian survey reported a high prevalence of hypovitaminosis D among postmenopausal women (68%) (^28^).

Our study showed that all anthropometric variables improved across VitD quartiles. Studies have demonstrated that low VitD concentrations increase parathyroid hormone (PTH) levels (^29^). Elevated PTH levels increase calcium influx into adipocytes, leading to fat accumulation and weight gain (^30^). Additionally, significant increases in adipose tissue can sequester fat-soluble VitD from the bloodstream, subsequently reducing its circulating levels (^31^). Other studies have also found that higher VitD levels are negatively correlated with body mass index, body weight, and body fat percentage in postmenopausal women and other populations (^28^,^30^,^32^). Furthermore, some researchers have reported that VitD supplementation can improve these anthropometric parameters. A clinical trial involving 77 women with overweight or obesity concluded that 12 weeks of VitD supplementation was associated with a reduction in body fat mass (^33^). Although further research is needed, a systematic review and meta-analysis suggested that VitD supplementation offers a potential therapeutic option for weight loss (^30^).

Regarding lipid profiles, our study showed that parameters progressively improved from the first to the fourth quartile of VitD. Participants with VitD levels above 30 ng/mL (Q4) had significantly lower triglyceride and higher HDL-c levels than those with serum VitD concentrations below 21 ng/mL (Q1). Therefore, our results suggest that adequate VitD may be associated with a favorable lipid profile and a reduced risk of cardiovascular disease. VitD plays a crucial role in regulating calcium and PTH metabolism (^29^). Adequate serum VitD levels enhance the intestinal absorption of calcium, thereby reducing fatty acid absorption (^34^). Moreover, elevated VitD levels prevent the activation of PTH, which normally inhibits lipolysis, leading to increased lipolysis and reduced triglyceride levels (^35^,^36^). Additionally, VitD may affect lipoprotein metabolism by reducing triglyceride synthesis and secretion in the liver, culminating in decreased triglyceride and VLDL-c levels and an increased HDL-c levels (^36^). In a study involving Polish postmenopausal women, VitD was similarly associated with increased HDL-c and decreased total cholesterol and LDL-c (^37^). Furthermore, other studies have linked VitD deficiency to hyperlipidemia and MetS (^28^,^36^).

In this study, although the statistical analysis did not reveal significant differences, participants with higher VitD levels exhibited lower fasting blood glucose levels. VitD was also significantly associated with lower insulin levels and reduced insulin resistance. Several mechanisms have been proposed to explain the role of VitD in glucose metabolism: (^1^) direct stimulation of insulin secretion via the VitD receptor on pancreatic beta cells; (^2^) reduction of systemic inflammation, which subsequently mitigates insulin resistance; and (^3^) improvement of peripheral insulin sensitivity mediated by VitD receptors in the liver and skeletal muscles (^9^). Other studies have evaluated the relationship between VitD and glycemic levels in postmenopausal women as well. A review concluded that lower VitD levels correlated with higher glucose concentrations in participants aged 35-74 years (^15^). Moreover, Schmitt and colleagues (^28^) evaluated a cohort of Brazilian women and demonstrated that participants with hypovitaminosis D had higher levels of total cholesterol, triglycerides, insulin, and HOMA-IR than those with adequate VitD status. Conversely, another review concluded that there is insufficient evidence to support the use of VitD supplementation for managing insulin resistance and diabetes mellitus (^10^). Thus, further studies are needed to better understand how VitD influences carbohydrate metabolism in both the general population and postmenopausal women.

Some studies have reported that low VitD levels contribute to the inadequate activation of the renin-angiotensin system and increased blood pressure (^38^,^39^). These effects tend to be more prominent in postmenopausal women, as hypoestrogenism also increases blood pressure (^18^). However, in our study, we did not find significant differences in blood pressure according to the quartiles of VitD. The median systolic and diastolic blood pressure values of the participants were below or very close to the reference range, which may have hindered the detection of blood pressure variations across VitD levels. It is also pertinent to highlight that 38.7% of the women participating in this study reported having hypertension and using antihypertensive drugs. Because many participants had their blood pressure controlled by medication, this control may have masked potential associations. Similarly, a clinical trial did not find an association between VitD levels and blood pressure in American postmenopausal women (^40^). Despite this, we recommend further studies to identify the role of VitD in the blood pressure of middle-aged women.

MetS is a group of metabolic risk factors that includes abdominal obesity, dyslipidemia, hypertension, and hyperglycemia. In perimenopausal and postmenopausal women, MetS is more prevalent because most of its components are adversely affected by reproductive aging. Several studies have shown an inverse relationship between serum VitD levels and MetS, diabetes, and insulin resistance in the general population (^41^). Nevertheless, data regarding postmenopausal women are limited and contradictory.

Recent meta-analyses of randomized controlled trials have reported conflicting results. One meta-analysis found that VitD supplementation improved glucose metabolism but had no significant effect on body composition or obesity parameters in postmenopausal women (^42^). Another review concluded that the effects of VitD supplementation on lipid parameters, specifically reductions in LDL-C and increases in HDL-C and total cholesterol, were clinically negligible (^43^). Together, these findings underscore the need for continued research and suggest that individualized VitD dosing strategies may be required, depending on the specific metabolic outcomes targeted in postmenopausal women.

In our study, we found that postmenopausal women with VitD levels below 21 ng/mL (Q1) had 2.27 times higher odds of having MetS compared with women with levels above 30 ng/mL (Q4). As demonstrated in our study and other surveys, VitD is associated with unfavorable changes in all parameters comprising MetS, which explains this result. A study evaluating 340 postmenopausal Thai women also showed that low VitD was associated with an increased frequency of MetS. Additionally, studies performed on postmenopausal Brazilian women showed that hypovitaminosis D was associated with an increased prevalence of MetS (^28^,^44^).

Researchers have recommended vitD levels between 30-60 ng/mL for specific groups, including patients aged >65 years, pregnant women, individuals with recurrent falls, fragility fractures, osteoporosis, secondary hyperparathyroidism, chronic kidney disease, or cancer, and individuals using medications that can potentially affect VitD metabolism (^45^). However, there is no specific reference range for vitD levels in postmenopausal women. Our study demonstrated better anthropometric and biochemical profile outcomes in postmenopausal women with VitD levels above 31 ng/mL. Similarly, we found the worst results in participants with VitD serum concentrations below 21 ng/mL. These findings suggest that maintaining serum VitD concentrations above 30 ng/mL may be beneficial for the metabolic health of postmenopausal women.

In this context, our results support the inclusion of serum VitD assessment in the routine health monitoring of postmenopausal women, particularly those with metabolic risk factors. Given the high prevalence of hypovitaminosis D and its association with adverse cardiometabolic profiles, strategies such as VitD supplementation, lifestyle modifications aimed at increasing sunlight exposure, and dietary guidance may serve as low-cost preventive measures (^4^,^26^). These interventions could help reduce the prevalence of MetS and improve overall metabolic health in this population.

This study has certain limitations. We did not assess dietary VitD intake or sun exposure, both of which are key determinants of serum VitD concentrations and might have contributed to individual variability in our findings. Although women who reported using VitD supplements were excluded, we did not control for the use of other medications that may affect VitD metabolism. Additionally, the cross-sectional design precludes causal inferences. Future longitudinal studies are needed to further investigate the potential protective role of VitD against MetS in postmenopausal women.

## CONCLUSION

This population-based study demonstrated an inverse association between serum VitD concentrations and the presence of MetS in postmenopausal women. Women with VitD levels below 21 ng/mL had more than twice the risk of developing MetS compared to those with adequate levels. These findings highlight the potential role of VitD as a biomarker of metabolic health and underscore the importance of routine monitoring in postmenopausal populations as a low-cost preventive strategy. Future longitudinal and randomized controlled studies are warranted to confirm these associations and to determine whether improving VitD status can effectively reduce the risk of MetS and related cardiometabolic complications.

## Data Availability

all data generated or analyzed during this study were produced by the authors. Datasets are available from the corresponding author upon reasonable request.
